# Silibinin-modified Hydroxyapatite coating promotes the osseointegration of titanium rods by activation SIRT1/SOD2 signaling pathway in diabetic rats

**DOI:** 10.1007/s10856-022-06684-1

**Published:** 2022-09-04

**Authors:** Zhou-Shan Tao, Hai-Sheng Wang, Tian-Lin Li, Shan Wei

**Affiliations:** 1 Department of Orthopedics, The First Affiliated Hospital of Wannan Medical College, Yijishan Hospital, No. 2, Zhe Shan Xi Road, Wuhu, 241001 Anhui P.R. China; 2 Key Laboratory of Non-coding RNA Transformation Research of Anhui Higher Education Institution (Wannan Medical College), No. 2, Zhe Shan Xi Road, Wuhu, China; 3 School of Mechanical Engineering, Anhui Polytechnic University, Wuhu, 241000 P.R. China; 4 Additive Manufacturing Institute of Anhui Polytechnic University, Anhui Polytechnic University, Wuhu, 241000 P.R. China

## Abstract

The purpose of this study is to investigate the role of Silibinin (SIL)-modified Hydroxyapatite coating on osseointegration in diabetes in vivo and in vitro and explore the mechanism of osteogenic differentiation of MC3T3-E1. RT-qPCR, Immunofluorescence, and Western blot were used to measure the expression level of oxidative Stress Indicators and osteogenic markers proteins. Moreover, CCK-8 assay was conducted to detect cell viability in hyperglycemia. Alizarin red staining and alkaline phosphatase staining were used to examine osteogenic function and calcium deposits. The diabetic rat model receive titanium rod implantation was set up successfully and Von-Gieson staining was used to examine femoral bone tissue around titanium rod. Our results showed that intracellular oxidative stress in hyperglycemia was overexpressed, while FoxO1, SIRT1, GPX1, and SOD2 were downregulated. SIL suppressed oxidative stress to promote osteogenic differentiation. Additionally, it was confirmed that SIL promoted osteogenic differentiation of MC3T3-E1 and obviously restored the osseointegration ability of diabetic rats. Further study indicated that SIL exerted its beneficial function through activation SIRT1/SOD2 signaling pathway to restore osteoblast function, and improved the osseointegration and stability of titanium rods in vivo. Our research suggested that the SIL-modulated oxidative Stress inhibition is responsible for the activation of the process of osteogenic differentiation through activation SIRT1/SOD2 signaling pathway in hyperglycemia, providing a novel insight into improving prosthetic osseointegration in diabetic patients.

**Figure:**
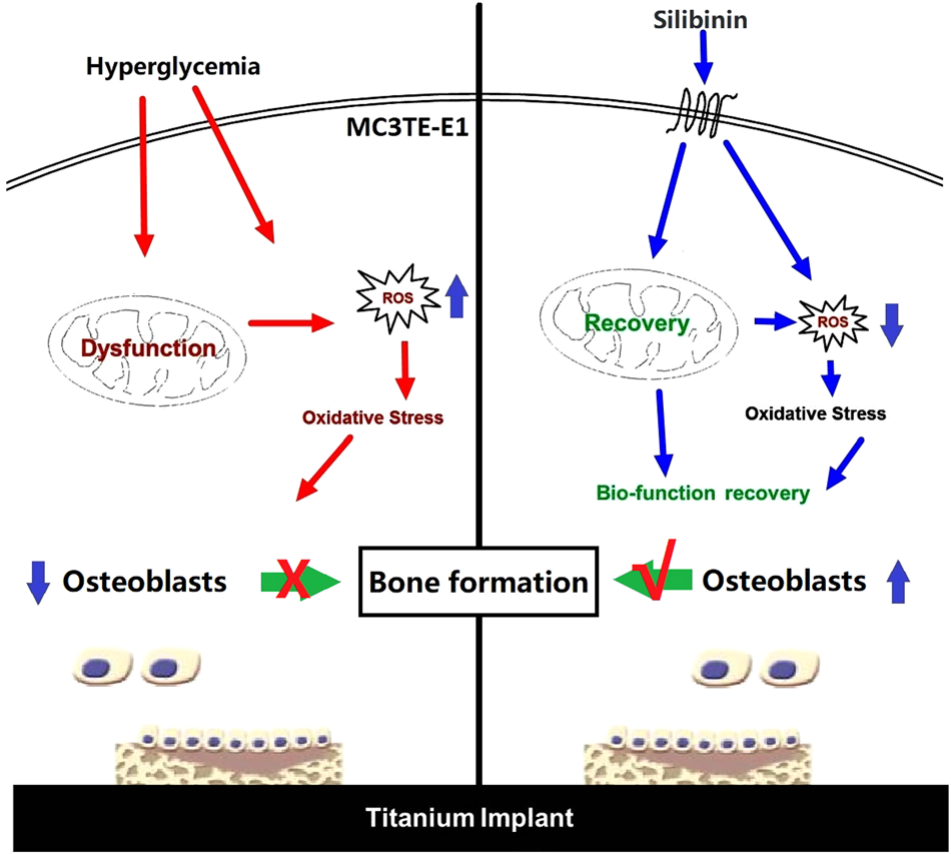
Hyperglycemia impaired the activity and function of MC3T3-E1 and inhibits bone formation by up-regulating intracellular ROS levels through inhibition of SIRT1/SOD2 signaling pathway. Local administrator SIL can improve the activity and function of osteoblasts and enhance osseointegration by reducing intracellular ROS through activation of SIRT1/SOD2 signaling pathway in DM rat models.

Hyperglycemia impaired the activity and function of MC3T3-E1 and inhibits bone formation by up-regulating intracellular ROS levels through inhibition of SIRT1/SOD2 signaling pathway. Local administrator SIL can improve the activity and function of osteoblasts and enhance osseointegration by reducing intracellular ROS through activation of SIRT1/SOD2 signaling pathway in DM rat models.

## Introduction

Total joint replacement has been proven to be a highly effective treatment for severe end-stage arthritis and joint dysfunction, because the procedure restores function, reduces pain and increases quality of life and satisfaction [1]. With increase in prolonged life expectancy and metabolic bone diseases, the number of joint replacement surgery in demand is expected to increase markedly [2]. With advances in technology and surgical techniques, the incidence of aseptic loosening or other causes of aseptic failure, which may also require implant revision surgery, has increased in recent years [3]. Diabetes mellitus (DM) is a type of metabolic disease characterized by chronic hyperglycemia [4]. In recent years, studies have confirmed that patients with DM have a higher incidence of prosthesis loosening [5]. Due to the loss of bone at the implant surface, insufficient bone formation and untimely and adequate osseointegration caused by diabetes complicated with secondary osteoporosis, prostheses in diabetic patients are liable to undergo loosening [6, 7]. With the rapidly growing in prevalence, DM becoming an increasingly important global public health issue. How to reduce revision arthroplasty and improve the survival rate of artificial joints in diabetic patients with poor osseointegration has become a clinical problem now [7].

The pathological process and molecular mechanism of diabetic osteoporosis and development is not yet fully understood, and often, involves multiple influencing factors and environmental factors. At present, a large number of studies have agreed that hyperglycemia-induced oxidative stress which generates reactive oxygen species (ROS), plays an important role in the pathogenesis and progression of diabetic osteoporosis [8, 9]. The increased oxidative stress levels further worsen osteoporosis, and in turn, oxidative stress also reduces osteoblast activity and functional response.

Since osteoblasts play a central role in bone formation, the formation and activity of osteoblasts also seriously affects bone osseointegration. Therefore, reducing oxidative stress in vivo and osteoblasts in the diabetic state becomes a feasible solution to improve the successful and effective osseointegration of implants. Silibinin (SIL), a flavonolignan extracted from milk thistle (Silybum marianum), has exhibited considerable antioxidant stress activity and has been widely concerned [10]. In addition, hepatoprotective, anti-inflammatory, antioxidant and anti-cancer effects of SIL have been defined in various animal and in vitro models [11]. Furthermore, silibinin not only has excellent performance in the field of liver protection, but also plays an important role in bone health by promoting osteogenesis and increasing bone mass [12]. Our previous studies have reported that local application of silibinin can promote titanium rod osseointegration and repair of bone defects in osteoporotic rats induced by ovariectomy [9, 13]. Nonetheless, it is still unclear whether SIL can promote the osseointegration of titanium rods in diabetic rats.

In view of the above results, we propose the hypothesis that SIL can restore osteoblast function in a high glucose environment by virtue of its strong anti-oxidative stress ability, thereby restoring the ability of bone regeneration and osseointegration in diabetic rats. Therefore, in our current research, we explored the effect of Silibinin-modified Hydroxyapatite coating on osseointegration of titanium rods in a diabetic rat model, and initially explored possible mechanisms.

## Materials and methods

### Experimental animals and Silibinin-modified hydroxyapatite-coated titanium rods

Thirty female Sprague Dawley rats (210 and 240 g; age ∼3 months) were used in the present study. The rats were fed in a temperature-controlled environment with a 12-hour light/dark cycle and free access to water and a standard laboratory diet. All animal experiments were carried out in accordance with a protocol approved by the Institutional Animal Care and Use Committee of wannan medical college (Approval No. LLSC-2020-082).

The commercial titanium rods (TR, Zhejiang Guangci Medical Appliance Co., Ltd., Ningbo, China) with external diameter (1.2 mm) and length (20 mm) were used in vivo experimental of this study. HA coatings were prepared using the pulse electrodeposition process with a coating thickness of ~10 μm on the TR as our previously described [14, 15]. According to previous studies [13, 16], silibinin solution (0.12 g, 0.25 mmol) was dripped into HA-TR to complete Silibinin-modified hydroxyapatite-coated titanium rods (SHA-TR). SHA-TR and HA-TR were obtained after further lyophilization. Slectron microscope (FE-SEM, JSM-7500F; JEOL) and surface roughness tester (Mitutoyo SJ-400, Mitutoyo, Sakado, Japan) were used to observe phase composition and surface roughness of this coatings. SHA-TR was soaked in PBS solution (50 mL) at 37 °C. Thereafter, 1 mL of the release medium was taken out after 2, 4, 6, 8, 12, 14, 16, 18, 20, 22 day, and SIL release was determined by high performance liquid chromatography (HPLC, DGU-20A5R, Japan). SHA-TR and HA-TR were dissolved in DMSO to achieve a 100 mM stock solution, which was then diluted in plain DMEM for the required concentration (60 μM) for follow-up cell experiments [16].

### Influence of MC3TE-E1 cells viability, mineralization and ALP expression

MC3TE-E1 Cells(Purchased from Qingqi (Shanghai) Biotechnology Development Co., Ltd.) were used in all experiments to be seeded on the specimens with 1 × 10^4^ cells/ml and randomized to incubate with the following factors respectively: (1) normal milieu (Con); (2) diabetic milieu (DM); (3) diabetic milieu+ 60 μM SHA-TR leachate (SIL + DM; Sigma). DMEM containing 25 mmol/L glucose and 500 mmol/L BSA-conjugated palmitate (high glucose and fat) was used as the mimic milieu of type 2 diabetes [17] and labeled as DM.

Cell Counting Kit-8 (CCK-8), Alizarin Red S (ARS) staining and Alkaline phosphatase (ALP) staining were used to determine the effects of SIL and high glucose on MC3TE-E1 Cells biological characteristics including changes in the activity, function, intracellular ROS levels and the expression of related proteins.

Briefly, MC3TE-E1 was seeded in a 96-well plate with a density of 1 × 10^4^ cells per well and treated with normal saline, high glucose and 60 μM SHA-TR leachate +high glucose as mentioned above for 24 h. Subsequently, the wells of different groups were added with 10-μl CCK-8(Med Chem Express LLC; Monmouth Junction, NJ, The USA) solution for 2 more hours and evaluated through a Multiskan Go Microplate Spectrophotometer (Thermo Fisher Scientific).

Meanwhile, the changes of total intracellular reactive oxygen species (ROS) contents for MC3TE-E1 under different interventions were measured by the fluorescent probe 2’, 7’-dichlorofluorescin diacetate (DCFDA). Immunofluorescence analysis including SIRT1 and SOD2 staining were used to quantify the expression of regulatory factors of MC3TE‐E1 as stated above.

For ARS staining and ALP staining, MC3TE-E1 was seeded in a 96-well plate with density of 1 × 10^4^ cells per well and treated with osteogenic medium (complete α-MEM containing 1 nM dexamethasone, 50 μM ascorbic acid, and 20 mM β-glycerophosphate) [18] before reaching over 80% confluence. Next, the MC3TE-E1 cells receive interventions with different treatment options as mentioned above. After treatment for 14 days, osteogenesis was assessed using Alkaline phosphatase (ALP, Beyotime Institute of Bio-technology; Jiangsu, China) staining. After treatment for 21 days, osteogenic differentiation and cell mineralization was detected using Alizarin Red S (ARS) solution (Solarbio Science & Technology). The results ALP staining and RES staining were determined by microscopy and quantified with Image Pro Plus 6.0 software.

### Western blot

Whole cell extracts of MC3TE-E1 for western blotting were prepared after the indicated treatment for 3 days as previously described [19]. 20 μg protein from total cell lysate was prepared using PRO-PREPTM protein extraction solution (Boca Scientific Inc., Boca Raton, FL) and carried out subsequent electrophoresis, according to the manufacturer’s instruction. The primary antibodies against the following proteins: FoxO1 (Abcam, ab52857, 1:1000), SIRT1 (Abcam, ab189494, 1:1000), human catalase (CAT, Abcam, ab130029, 1:1000), glutathione peroxidase (GPX1, Abcam, ab108427, 1:1000), superoxide dismutase 2 (SOD2, Abcam, ab252426, 1:1000). Expression levels of the target protein were normalized against glyceraldehyde 3-phosphate dehydrogenase (GAPDH) (Boster, Wuhan, China, 1:2000) levels in each sample. The following day, HRP-conjugated goat anti-rabbit, which used as secondary antibodies, was purchased from Santa Cruz Biotechnology (Santa Cruz, CA) and the results were detection and analysis by using an iBrightCL1000 (Invitrogen, Carlsbad, CA).

### Animal experiment

Diabetic rat model for this study was induced by receiving a single 35 mg/kg streptozotocin (STZ, Sigma Aldrich) injection (dissolved in 0.1 M citrated buffer, pH 4.5). Only rats with blood glucose levels >300 mg/dL were employed as a successful model of diabetic rats according to previous reports [20]. The rats were randomly divided into 3 groups: control group (Con, *n* = 10), diabetic rat group (DM, *n* = 10) and diabetic rat receiving local silibinin treatment group (SIL + DM, *n* = 10). The HA-TR and SHA-TR were inserted femoral cavity bilaterally in all animals from Con group, DM group and SIL + DM group, respectively, as previously described [21]. Twelve weeks after implantation, the rats were sacrificed to obtain blood and femurs containing titanium rods for subsequent testing.

### Micro-CT evaluation

After the execution of the experimental rats, the femurs containing titanium rods were taken bilaterally and soaked in paraformaldehyde. A micro-CT (SkyScan 1176)scanner was used for thin-layer scanning of bilateral femurs (imaging conditions: tube voltage, 90 kV; tube current, 88 μA; magnification, ×6.7; measurement time, 17 s; resolution, 30 μm; slice thickness, 240 μm; and slice spacing, 240 μm) as previously described [22, 23]. The scanning area was a distance of 2 mm proximal to the growth plate in the femoral metaphysis. The volume of interest (VOI) with the central 250-μm-diameter region of the surface of titanium rod. 3D multimodel software was utilized following the scanning process to generate the specimen data for the following indicators: bone mineral density (BMD), bone volume fraction (BV/TV), trabecular number (Tb.N), trabecular thickness (Tb.Th), trabecular separation (Tb.Sp), the mean connective density (Conn.D) in VOI regions.

### Mechanical test

The femurs undergo pull-out tests through the material testing system (Electron E1000, Instron, High Wycombe, UK). The left femur containing the titanium rod is first subjected to epiphyseal separation to expose the titanium rod for pull-out tests. Then these fixed specimens were subjected to pull-out tests by biomechanical machine through load-displacement curves to acquire mechanical parameters including the strength of fixation, energy failure and interface stiffness according to previous report [24].

### Histological evaluation and immunofluorescence analysis

The undecalcified femur specimens containing titanium rods were embedded in methyl methacrylate, and cut and ground to a thickness of 40–50 μm by using Saw Microtome Leica SP1600 (Leica, Wetzlar, Germany). Histological examination of undecalcified sections were performed by stained with Von-Gieson for light microscopy according to established methods [25, 26].

The titanium rods were removed and femur specimens without implant were decalcified with 10% EDTA (pH 7.4), dehydrated and embedded in paraffin prior to processing for SIRT1 and SOD2 staining. In brief, fresh bone sections were stained with individual primary antibodies to rats SIRT1 (Abcam, ab189494, 1:100) and SOD2 (Abcam, ab208156, 1:100), overnight at 4 °C. Subsequently, the secondary antibodies conjugated with fluorescence (Jackson Immuno Research, 415-605-166, 1:500; 315-605-003, 1:250) were used at room temperature for 1 h while avoiding light and observed under a confocal microscope (FLUOVIEW FV300, Olympus).

### Serum bone metabolism index and oxidative stress index detection

The serum concentrations of SOD2, MDA and TAC were determined using commercial biochemical kits from Nanjing Jiancheng Bioengineering Institute (Nanjing, China). The serum concentrations of osteocalcin (OC) and tartrate-resistant acid phosphatase (TRAP) determined by ELISA kits from Beijing Fang cheng Biotechnology Company (Beijing, China). All experiment steps and methods are according to the manufacturer’s instructions.

### Statistical analysis

The data were collected and reported as the mean ± standard deviation (SD) for the final result of each experiment. The *t* test and one-way analysis of variance (ANOVA) test were used to test differences between different groups. IBM SPSS Statistics 21.0 software (IBM SPSS Inc., Chicago, IL) was used for analyses. *P* values of <0.05 were considered statistically significant.

## Results

### characterization of HA-TR and SHA-TR

**Fig. 1:**
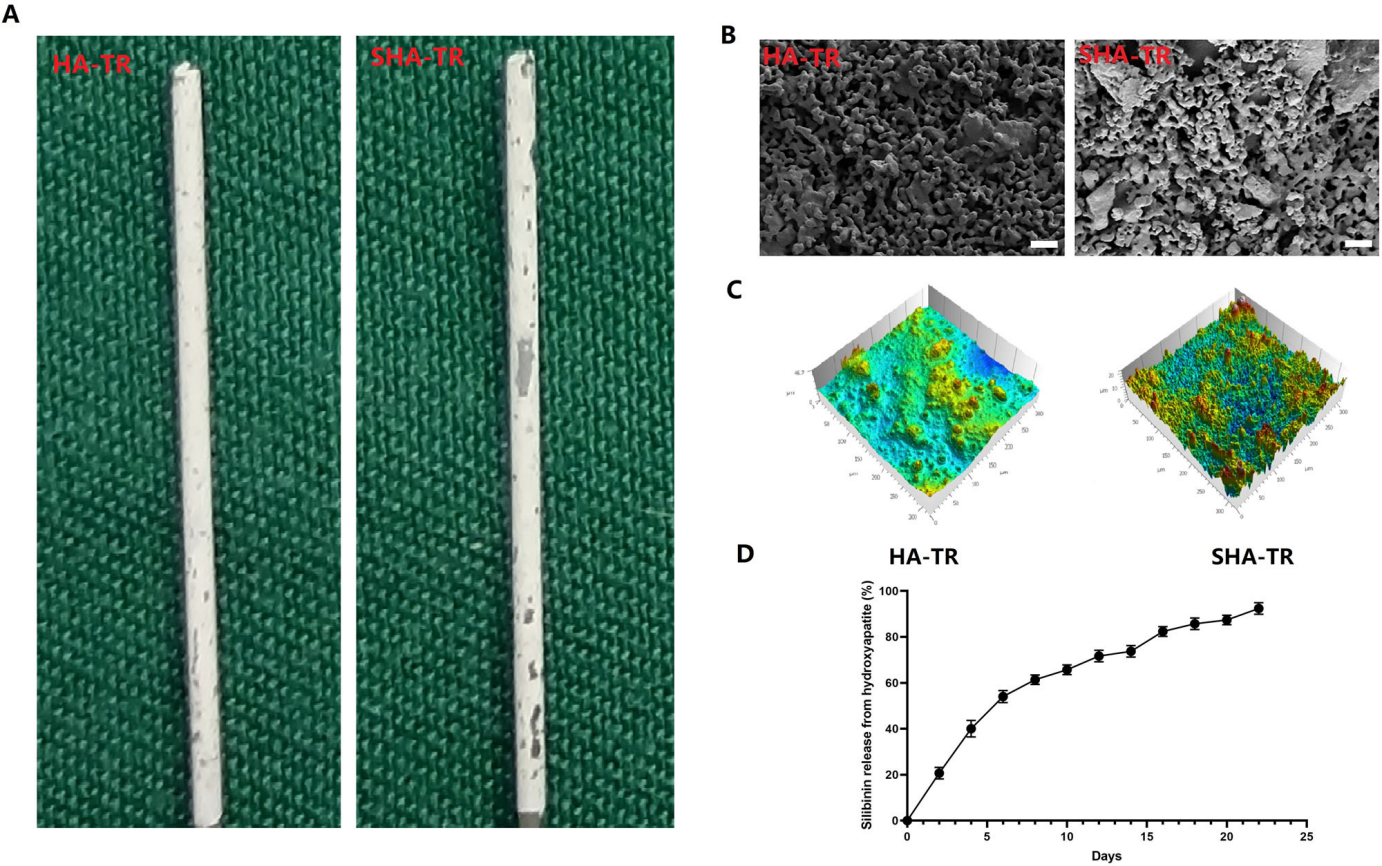
The surface characteristics and SIL Release characteristics of HA-TR and SHA-TR. **A** Representative pictures of general view of HA-TR and SHA-TR; **B**: Representative pictures of higher magnification of surface characterizations of HA-TR and SHA-TR, Scale bar: 10 μm; **C**: Representative pictures of surface roughness of HA-TR and SHA-TR; **D**: Percentage of released SIL relative to total SIL loaded into coatings

General view and higher magnification of surface characterizations of HA-TR and SHA-TR is shown in Fig. 1A, B. The crystallite and roughness in SHA coatings seemed obviously higher than that in HA coatings from SEM micrographs. The SIL showed relatively rapid release in the 10 days, followed by a steady release phase (Fig. 1D). Due to the addition of SIL, the structure of HA is changed, and an increase in surface roughness occurs.

### The changes of MC3TE-E1 cells viability, mineralization, ALP and target protein expression in a hyperglycemia environment

**Fig. 2:**
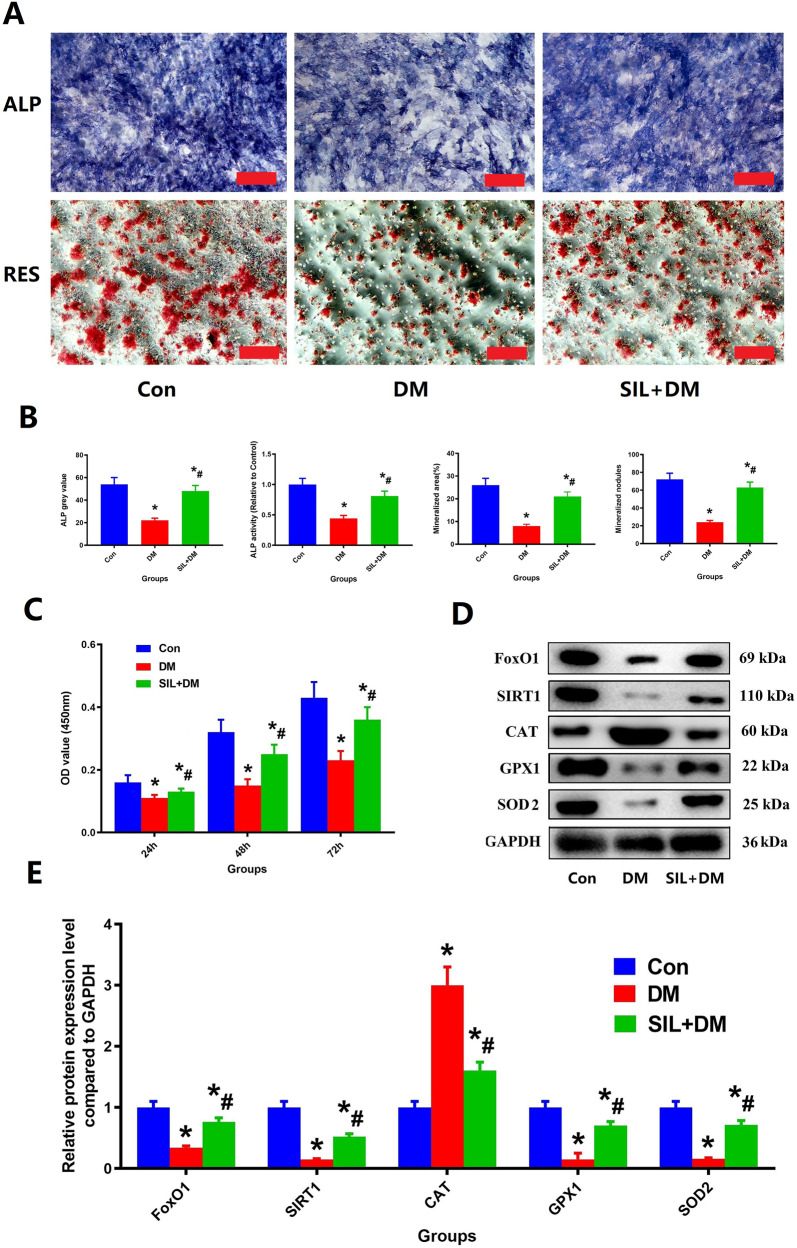
SIL therapy could greatly enhance the biological activity of MC3TE-E1 in a hyperglycemia environment. **A** Representative pictures of ALP staining and RES staining of MC3TE-E1 under different intervention conditions; **B**: The quantification of mineralized nodules, mineralized area, ALP activity and ALP gray value; **C**: The results of CCK-8 of MC3TE-E1 under different intervention conditions; **D**: Representative pictures of WB results of MC3TE-E1 under different intervention conditions; **E**: The quantitative results of related protein expression of WB. Scale bar: 50 µm. *N* = 5 specimens/group. *Vs. Con group, *P* < 0.05, #Vs. DM, *P* < 0.05

Figure 2A clearly shows us that a hyperglycemia environment can significantly reduce the ALP expression and calcification capacity of MC3T3-E1 cells. However, SIL intervention therapy can significantly increase the ALP expression and calcification capacity of MC3T3-E1 cells in a hyperglycemia environment. The quantitative results of ALP expression and calcification for each group are shown in Fig. 2B. The quantification of mineralized nodules, mineralized area, ALP activity and ALP gray value were greater in the SIL + DM group compared with the DM group (*P* < 0.05).

Figure 2C, D clearly shows us that a hyperglycemia environment can significantly reduce the viability and related protein expression including FoxO1, SIRT1, GPX1 and SOD2 of MC3T3-E1 cells, whereas CAT increased obviously were observed in DM group. However, SIL intervention therapy can significantly increase the viability and FoxO1, SIRT1, GPX1 and SOD2 expression of MC3T3-E1 cells. Besides, the levels of CAT decreased obviously were observed in SIL + DM group. The quantification of CCK-8, FoxO1, SIRT1, GPX1 and SOD2, CAT shown obvious restore in the SIL + DM group compared with the DM group (*P* < 0.05, Fig. 2C, E).

**Fig. 3:**
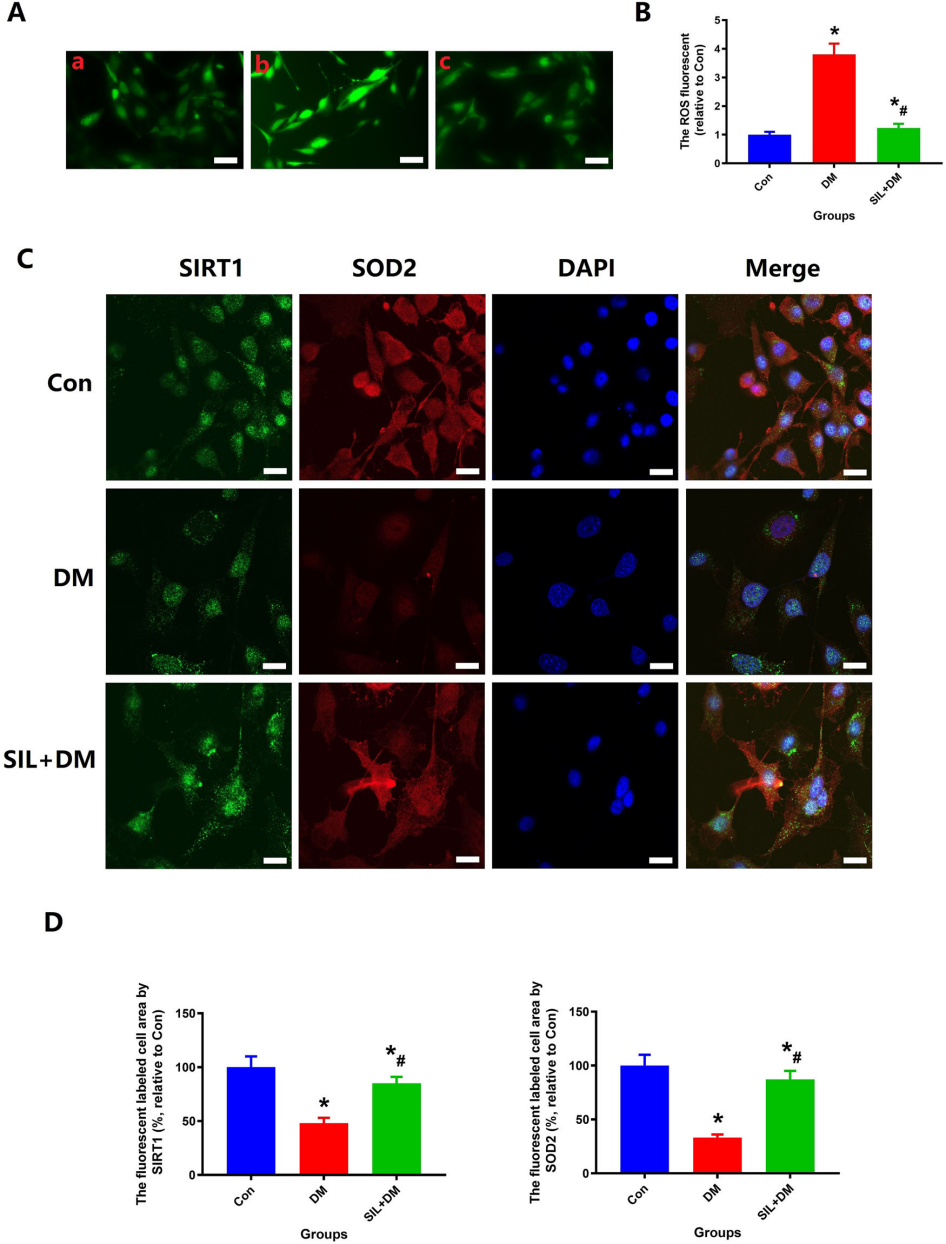
SIL therapy could reduce ROS levels and SIRT1 and SOD2 expression in MC3TE-E1 cells in a hyperglycemia environment. **A**: Representative pictures of Intracellular ROS of MC3TE-E1 under different intervention conditions, Scale bar: 25 µm; **B**: Quantitative results of intracellular ROS of MC3TE-E1; **C**: Representative pictures of SIRT1 and SOD2 expression of MC3TE-E1 under different intervention conditions, Scale bar: 25 µm; **D**: Quantitative results of SIRT1 and SOD2 expression of MC3TE-E1; *N* = 5 specimens/group. *Vs. Con group, *P* < 0.05, #Vs. DM, *P* < 0.05

Immunofluorescence results show a hyperglycemia environment can significantly reduce SIRT1 and SOD2 expression and increase ROS level as shown in Fig. 3A, C, and SIL intervention therapy can significantly increase SIRT1 and SOD2 expression, and decrease ROS level. The quantification of ROS, SIRT1 and SOD2 expression were restored in the SIL + DM group compared with the DM group (*P* < 0.05). These results indicate that SIL intervention reversed the inhibition of hyperglycemia environment on MC3T3-E1 cells, and enhance the ability of cells to resist oxidative stress by activating the SIRT1/SOD2 signaling pathway.

### Animal

A total of 3 rats died in this study due to anesthesia accidents and postoperative infections, of which 1 belonged to the DM group and 2 belonged to the SIL + DM group. Finally, a total of 27 rats completed the experiment.

### Micro-CT assessment of osseointegration

**Fig. 4:**
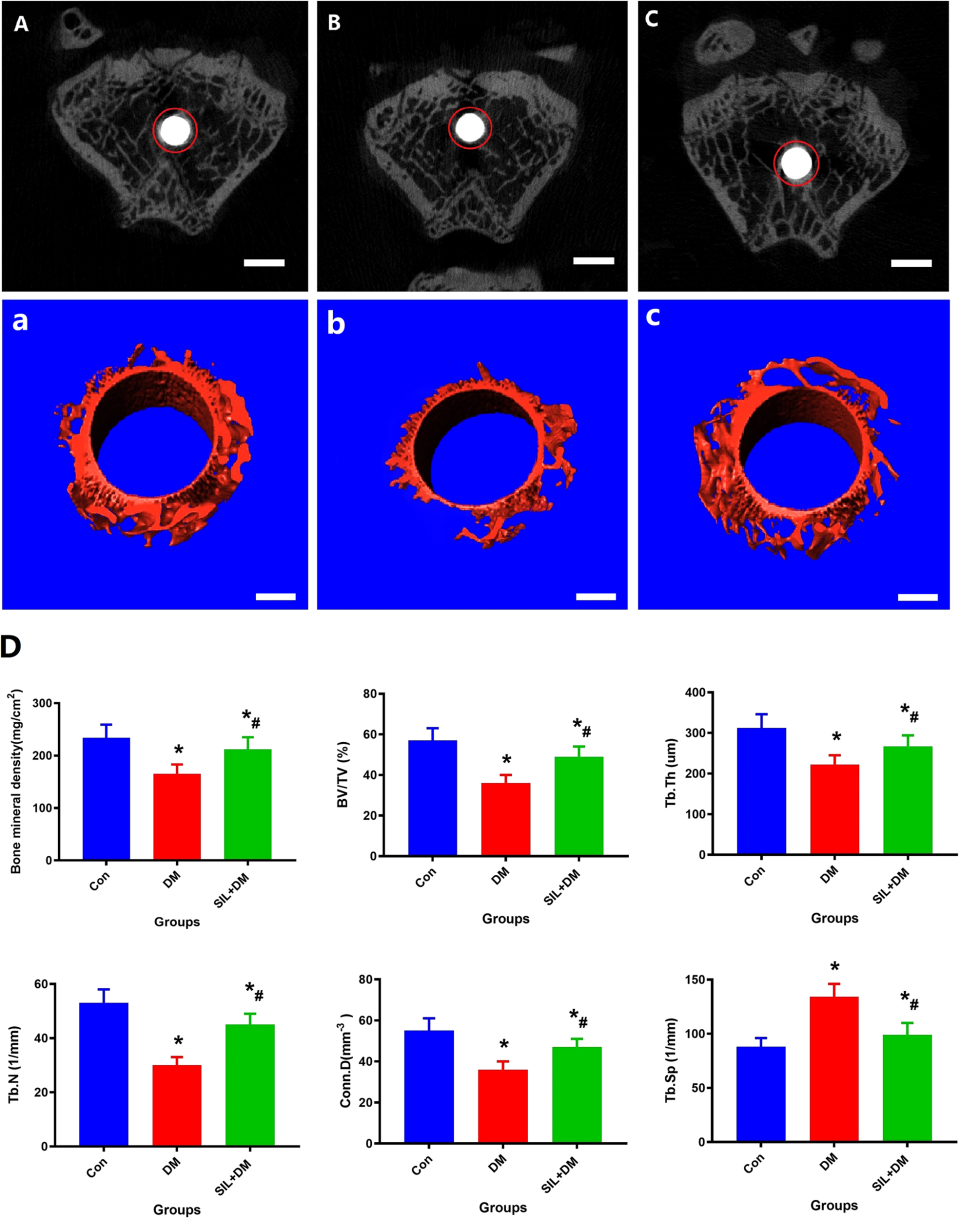
Micro-CT scan and 3D reconstruction clearly shows that local administrator SIL can significantly restore the poor capacity for titanium rods osseointegration in diabetic rats. **A**, a: Con group; **B**, b: MD group; **C**, c: SIL + DM group. **D**: The quantitative results of bone microscopic parameters around the titanium rod include BMD, BV/TV, Tb. Th, Tb. N, Conn. D and Tb. Sp. Scale bar: 100 µm. *N* = 5 specimens/group; *Vs. Con group, *P* < 0.05, #Vs. DM, *P* < 0.05

The reconstructed three-dimensional (3D) and 2D scan images of new bone in the VIO eara are presented in Fig. 4, and quantitative microstructure parameters were calculated including BV/TV, Tb.Th, Tb.N, Tb.Sp, Conn.D and BMD. At 12 weeks, the BMD, BV/TV, Tb.Th, Tb.N, and Conn.D of DM rats were all lower than the Con group (*P* < 0.05), while Tb.Sp was higher than the Con group (*P* < 0.05). Moreover, in the DM rats receiving SIL therapy, the BMD, BV/TV, Tb.Th, Tb.N, and Conn.D were significantly lower than the DM group (*P* < 0.05), and Tb.Sp was significantly higher than the DM group (*P* < 0.05). In addition, the capacity for bone repair and bone mass around titanium rods was inhibited by hyperglycemia, while in the SIL + DM group, the ability of osseointegration was remarkably restored.

### Histological analysis

**Fig. 5:**
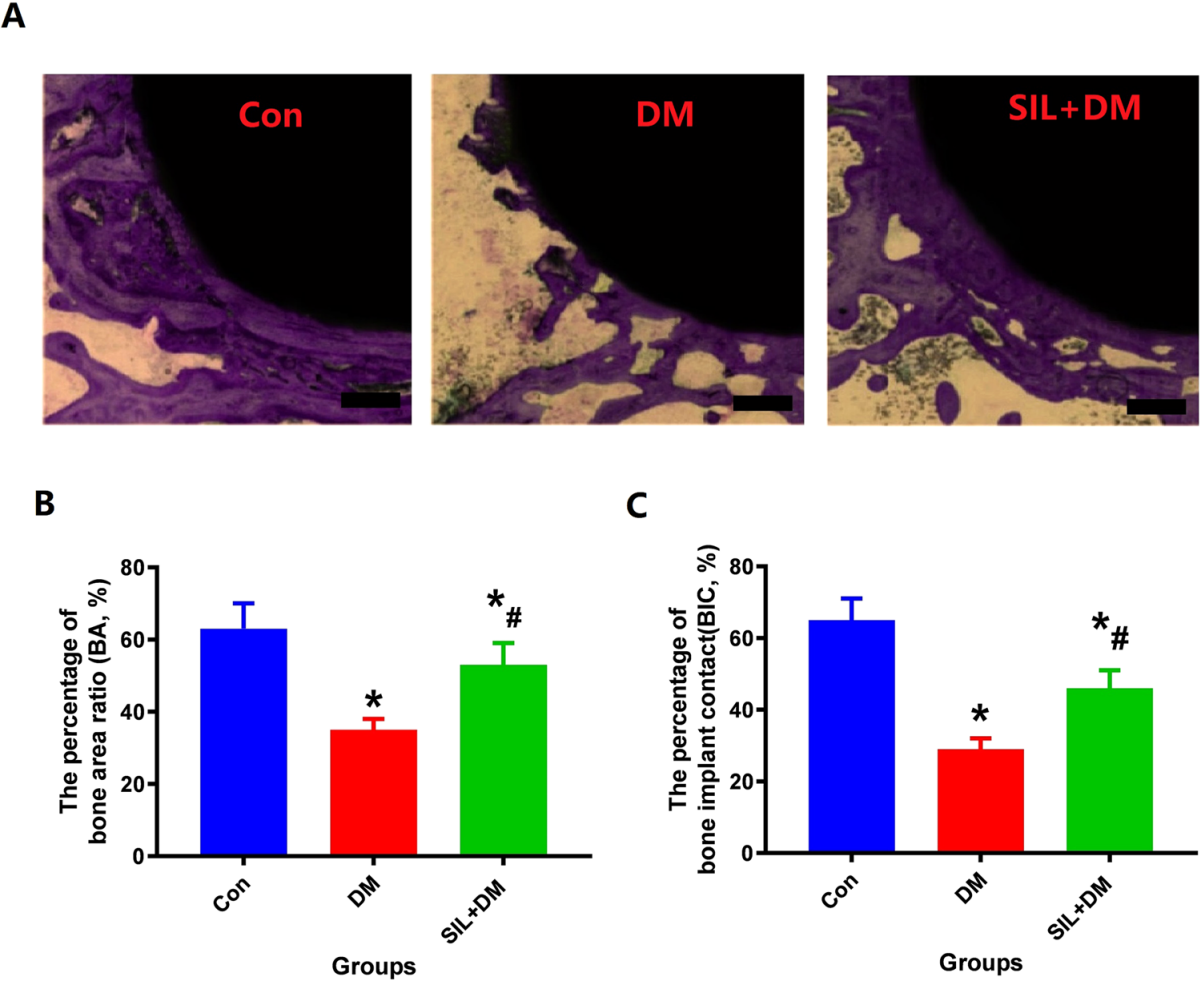
Local administrator SIL can significantly improve the bone formation around the titanium rod and increase the connection of bone tissue and internal plants in the diabetic rat model. **A**: Bone formation around titanium rods under different intervention conditions; Scale bar: 100 µm. **B**: The percentage of bone implant contact(BIC, %); **C**: The bone area ratio (BA, %); *N* = 5 specimens/group; error bars in the figure indicate SD. *Vs. Con group, *P* < 0.05, #Vs. DM, *P* < 0.05

Histological images with Von-Gieson staining staining of undecalcified sections are shown in Fig. 5. In the DM treatment group, fewer bone tissue contact with titanium rods were observed compared to the Con group. Besides, in the SIL + DM group, the areas around titanium rods contained large new bone tissue contact with titanium rods, and the more bone mass compared with the group DM. The quantitative results are represented by BIC and BA as shown in Fig. 5B, C. Compared with group Con, the significantly reduced BA% and BIC% were observed in DM group rats (*P* < 0.05). Besides, BA% and BIC% increased significantly after SIL treatment. Histological results show that local treatment with silibinin could increase bone tissue formation around titanium rods and promote the connection between bone tissue and titanium rods.

### Immunofluorescence analysis

**Fig. 6:**
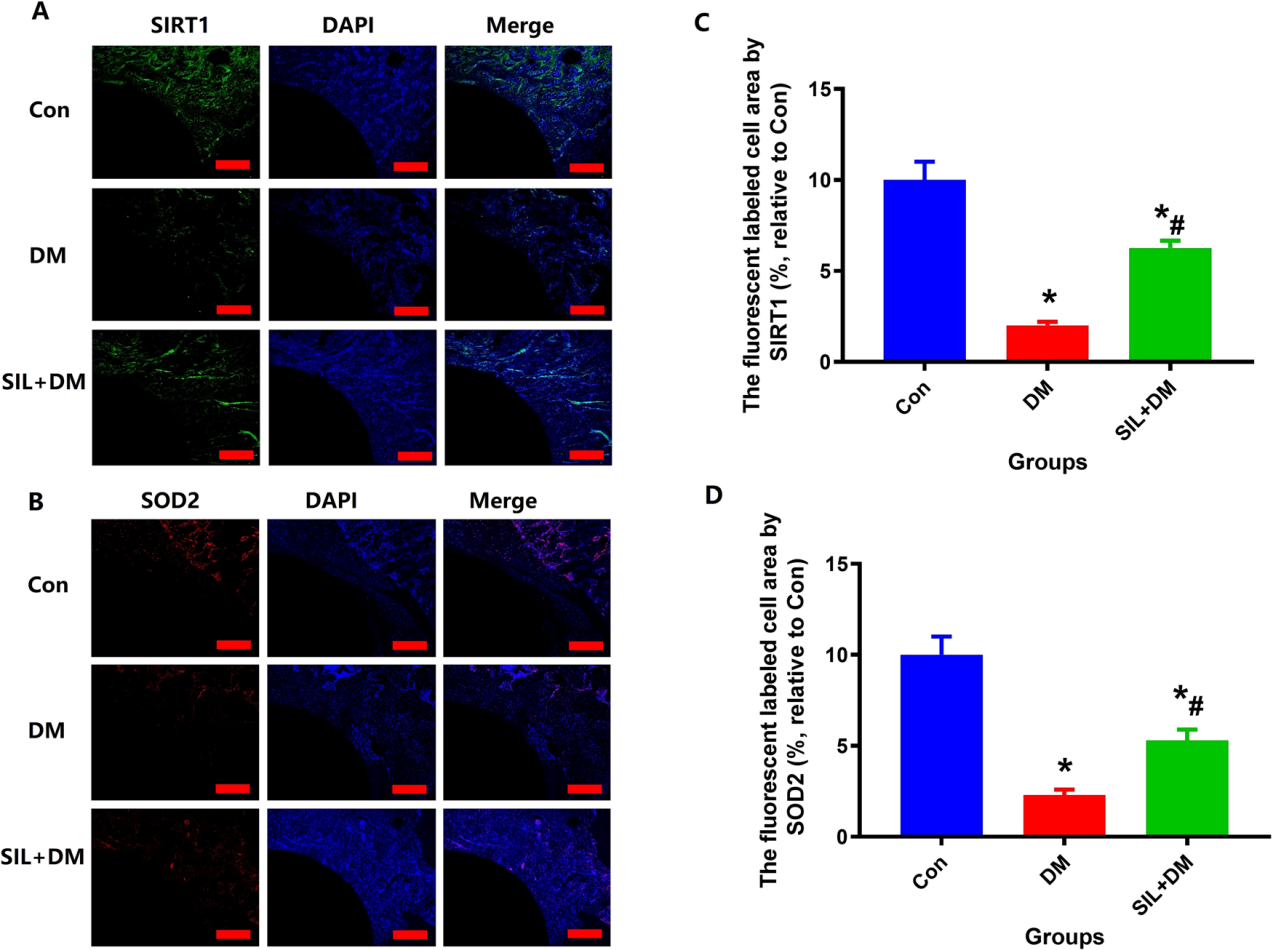
Local administrator SIL can significantly improve the expression of SIRT1 and SOD2 around titanium rod in diabetic rat model. Representative pictures of SIRT1 (**A**) and SOD2 (**B**) expression in bone tissue around titanium rod measured by immunofluorescence; The quantitative results of SIRT1 (**C**) and SOD2 (**D**) were expressed as fluorescently marked bone tissue area. Scale bar: 25 µm. *N* = 5 specimens/group. *Vs. Con group, *P* < 0.05, #Vs. DM, *P* < 0.05

The fluorescently labeled area of the Con group was almost filled with immunofluorescence for SIRT1 and SOD2, while large amounts of fluorescently labeled bone tissue was found in the SIL treatment group, but it was difficult to find immunofluorescence in the DM group (Fig. 6A, B). Compared to groups DM, SIL treatment shows the higher protein expression with SIRT1 and SOD2 (*P* < 0.05, Fig. 6C, D). Immunofluorescence l results show that local therapy with SIL showed positive effects on SIRT1 and SOD2 expressions in the diabetic condition.

### Biomechanical analysis

**Fig. 7:**
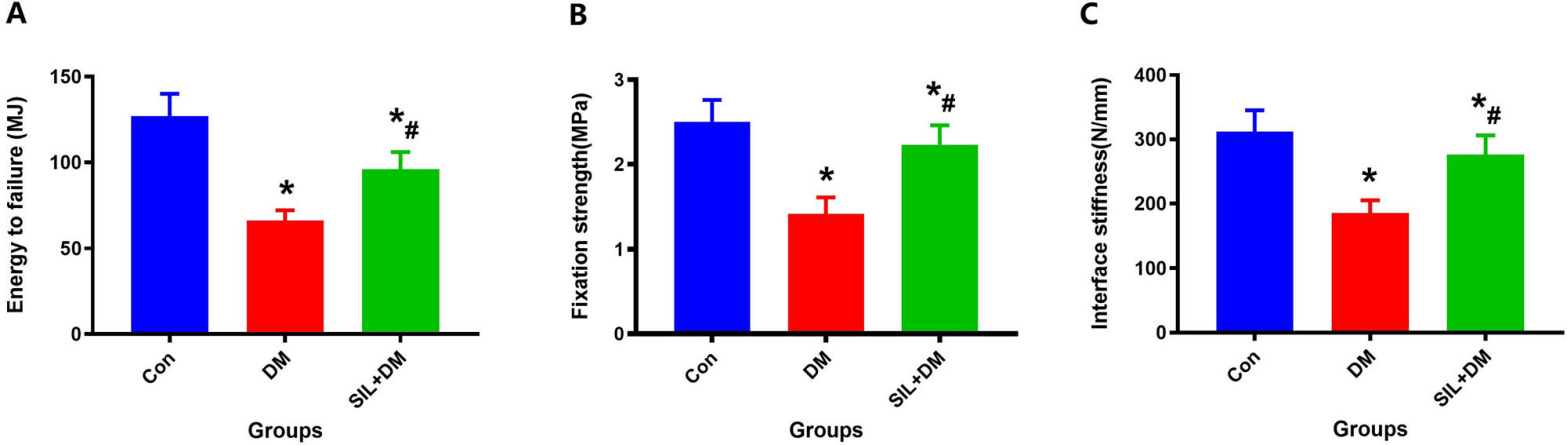
Local therapy with SIL can greatly enhance the mechanical pull-out data including energy to failure (**A**), fixation strength (**B**) and interface stiffness (**C**) of the titanium rod in the diabetic rat model; *n* = 5 specimens/group; *Vs. Con group, *P* < 0.05, #Vs. DM, *P* < 0.05

The results of the three groups of biomechanical experiments are presented as fixation strength, interface stiffness and energy to failure, as shown in Fig. 7. At 12 weeks post-intervention, results clearly exhibited diabetes can markedly weaken fixation strength, interface stiffness, energy to failure of the titanium rod compared with the Con group (*P* < 0.05). Moreover, SIL can notable improve the data of biomechanical parameters for the titanium rod compared with the DM group (*P* < 0.05). These results indicate that local treatment with SIL can obviously enhance the stability of the titanium implant in the marrow cavity of diabetic rats.

### Analysis of oxidative stress indicators and bone metabolism indexs

**Fig. 8:**
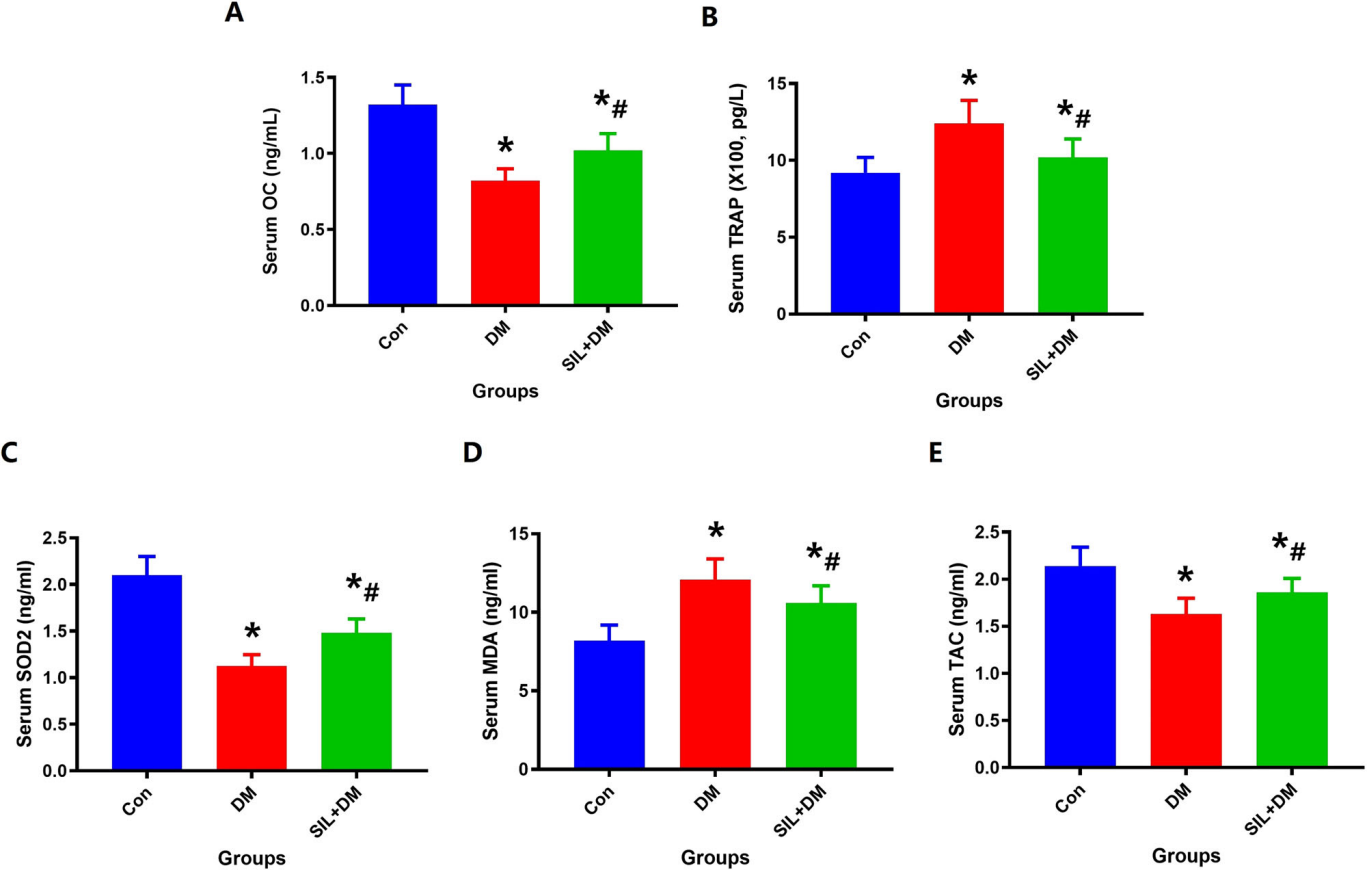
Local therapy with SIL can significantly reverse the imbalance of bone metabolism and oxidative stress in the diabetic rat model; **A**: Serum OC level; **B**: Serum TRAP level; **C**: Serum SOD2 level; **D**: Serum MDA level; **E**: Serum TAC level; *N* = 5 specimens/group. *Vs. Con group, *P* < 0.05, #Vs. DM, *P* < 0.05

The results of bone metabolism indicators and oxidative stress indicators were detected at the end of the experiment as shown in Fig. 8. Compared with the Con group, serum OC, TAC and SOD 2 decreased significantly while the level of TRAP and MDA increased obviously in diabetic rats. Compared with the DM group, serum OC, TAC and SOD2 increased obviously after SIL treatment, while TRAP and MDA decreased significantly. These findings indicate that local therapy with SIL can reverse the imbalance of bone metabolism and oxidative stress in DM rats.

## Discussion

Nowadays, long-term survival of joint prostheses in diabetic patients is still an enormous challenge to clinicians, owing to long-term hyperglycemia impairs bone formation compared with the nnormoglycemia. A lot of previous studies have identified that SIL has a substantial protective effect on bone mass by inhibiting oxidative stress. However, there are only limited reports on the effects of local application SIL on osseointegration in diabetes. In this study, SIL-modified HA-coated titanium rods were implanted into the femur of diabetic rats and their leaching solution was used to intervene osteoblast, and the effect of topical application of SIL on osteogenesis was observed in vitro and in vivo. The research confirmed that the local application of SIL can considerably restore the function of osteoblasts in a high glucose environment; meanwhile, it can enhance the osseointegration of titanium rods in diabetic rats.

As people’s living habits change, the higher prevalence of diabetes leads to an altered balance between bone resorption and bone formation and inadequate bone remodeling, resulting in bone loss [27]. Hyperglycaemia was shown to reduce bone resorption marker CTX and lower the rate of bone formation including osteocalcin, BALP and P1NP [28]. Moreover, hyperglycaemia also has been revealed to enhance advanced glycation end products, which disrupts the synthesis of collagen and bone matrix and lowers bone strength [29]. Despite evidence supporting its negative effect on mice osteoblast, hyperglycaemia has been further shown to inhibit osteoblast cell function in primary human osteoblasts [30]. However, the molecular mechanisms underlying diabetes-related bone loss are not fully understood. The present study showed diabetes markedly reduced bone formation around titanium rods and biomechanical parameters determined by Mciro-CT, histology and biomechanical experiment. Furthermore, a higher level of serum TRAP and lower level of serum OC were observed in the DM rats. In the present study, ALP staining and RES staining were used to assess osteogenic differentiation and function, and presented with lower ALP expression and fewer formation of mineralized nodules in hyperglycemia. Consistent with previous results [31, 32], the adverse effect of hyperglycaemia on osseointegration has also been certified in our current research.

As shown earlier [33, 34], silibinin has well-known antioxidant properties and effectively prevents oxidative stress and various complications caused by diabetes. It has been proposed that silibinin also restores mitochondrial potential and maintains mitochondrial homeostasis by scavenging ROS [35, 36]. In addition, studies have revealed that silibinin attenuates bone loss in DM rats and promotes bone formation in diabetes-related bone diseases [37, 38]. Despite the protection activities of silibinin on diabetes complications, whether silibinin can restore hyperglycemia-induced inhibition of osteoblast activity and impaired osseointegration remain unclear and need to further investigate. In the present study, we definitely identified that SIL could considerably inhibit the adverse effects of hyperglycemia on osteoblasts, including up-regulation of ALP expression, enhanced formation of mineralized nodules, and increased osteoblast activity. Animal experiments more intuitively revealed that the bone formation around the titanium rod was markedly strengthened after SIL therapy compared to the DM group, and the biomechanical parameters of the implants were significantly improved. According to the results, we preliminary believed that SIL could restore the osseointegration ability of diabetic rats to a certain extent.

Since hyperglycemia-mediated oxidative stress plays an important role in the impaired function of osteoblasts [39], we focused on exploring the changes in the expression of specific markers in osteoblasts, bone tissue and serum. As remarkable indexes, the levels of glutathioneylase 1 (GPX1), malondialdehyde (MDA), superoxide dismutase 2 (SOD2), total antioxidant capacity (TAC) can sensitive reflect the condition of oxidative stress in cells and in vivo [40, 41]. Sirtuin1 (SIRT1), an important member of SIRT family of nicotinamide adenine dinucleotide (NAD)-dependent deacetylases, regulate various processes of cellular metabolism and play an important role in bone loss [9]. As a downstream gene of SIRT1, forkhead transcription factor-1(FoxO1) can be activated by oxidative stress to promote the expression of downstream antioxidant genes to reduce oxidative stress to protect cell normal metabolism [42]. Numerous studies have shown that the inhibition of the SIRT1/SOD2 signaling pathway plays an important role in the damage to bone repair capacity caused by oxidative stress [9, 43]. WB and immunofluorescence disclosed that the oxidative stress markers protein including SIRT1, FoxO1, CAT, GPX1 and SOD2 in MC3T3-E1 of the DM group were markedly lower than those in the Con group. SIL promotes the expression of SIRT1, FoxO1, CAT, GPX1 and SOD2 of hyperglycemia Intervention of MC3T3-E1 by reducing intracellular ROS levels and restoring the activity and function of MC3T3-E1, similar to those from some researches [44, 45]. The results of bone tissue immunofluorescence and serum oxidative stress related indicators further confirmed this phenomenon. From these results, we believe that the level of oxidative stress increases in hyperglycemia, and SIL can restore the activity and function of osteoblasts by reducing oxidative stress, and improve the ability of osseointegration in diabetic rats.

In summary, our research results confirm that hyperglycemia impaired the activity and function of MC3T3-E1 and inhibits bone formation by up-regulating intracellular ROS levels through inhibition of SIRT1/SOD2 signaling pathway. Local administrator SIL can improve the activity and function of osteoblasts and enhance osseointegration by reducing intracellular ROS through activation of SIRT1/SOD2 signaling pathway in DM rat models. However, the lack of in vitro experiments on osteoclasts and the exploration of specific mechanisms made this study insufficient and prompted follow-up research to make up for these deficiencies.
